# Progress towards a glycoconjugate vaccine against Group A Streptococcus

**DOI:** 10.1038/s41541-023-00639-5

**Published:** 2023-03-28

**Authors:** Keira Burns, Helge C. Dorfmueller, Brendan W. Wren, Fatme Mawas, Helen A. Shaw

**Affiliations:** 1 Vaccine Division, Scientific Research & Innovation Group, MHRA, Potters Bar, UK; 2 Department of Infection Biology, London School of Hygiene and Tropical Medicine, London, UK; 3 Division of Molecular Microbiology, School of Life Sciences, Dow Street, Dundee, UK

**Keywords:** Conjugate vaccines, Bacterial infection, Policy and public health in microbiology

## Abstract

The Group A Carbohydrate (GAC) is a defining feature of Group A Streptococcus (Strep A) or *Streptococcus pyogenes*. It is a conserved and simple polysaccharide, comprising a rhamnose backbone and GlcNAc side chains, further decorated with glycerol phosphate on approximately 40% GlcNAc residues. Its conservation, surface exposure and antigenicity have made it an interesting focus on Strep A vaccine design. Glycoconjugates containing this conserved carbohydrate should be a key approach towards the successful mission to build a universal Strep A vaccine candidate. In this review, a brief introduction to GAC, the main carbohydrate component of Strep A bacteria, and a variety of published carrier proteins and conjugation technologies are discussed. Components and technologies should be chosen carefully for building affordable Strep A vaccine candidates, particularly for low- and middle-income countries (LMICs). Towards this, novel technologies are discussed, such as the prospective use of bioconjugation with PglB for rhamnose polymer conjugation and generalised modules for membrane antigens (GMMA), particularly as low-cost solutions to vaccine production. Rational design of “double-hit” conjugates encompassing species specific glycan and protein components would be beneficial and production of a conserved vaccine to target Strep A colonisation without invoking an autoimmune response would be ideal.

## Introduction

Vaccination is considered one of the most successful health interventions known to man^1^. Antibiotics and alternative therapeutics such as intravenous immunoglobulins are not suitable to control *Streptococcus pyogenes* or Group A Streptococcus (Strep A) infections at the population level or stop transmission within communities^2^. The emergence of antimicrobial resistance (AMR), along with the persistence of penicillin sensitivity, has led the WHO and CDC to both highlight vaccines as urgent safeguards against AMR^3,4^. Safe and efficacious Strep A specific vaccines are required for better control of Strep A related morbidity and mortality. Strep A is a major global pathogen, with disease manifestations ranging from Strep throat pharyngitis and impetigo to scarlet fever and invasive diseases such as toxic shock syndrome and necrotising fasciitis. These infections are often fast progressing and highly contagious, further emphasising the need for a vaccine. Invasive infections have high mortality and morbidity, and secondary diseases from autoimmune sequelae such as rheumatic heart disease (RHD) result in significant disease adjusted life years (DALYs), particularly in Low- and Middle-Income Countries (LMICs)^5^. In recent years there has further been a re-emergence of scarlet fever particularly noted in the UK and Europe, with particular concern in the current season with rising rates of sepsis associated with these infections^6,7^. There is therefore an urgent need globally to develop a vaccine against Strep A. With humans as the sole natural host there is also potential to eradicate the pathogen if transmission can be blocked.

Vaccine development against any pathogen is a complex and lengthy process, however Strep A vaccine development has had arguably a more complicated history with many challenges and hurdles. Strep A vaccine development has an official impeded status recognised by the WHO, due to many factors loosely relating to bacterial and host amongst others. First and foremost, Strep A is a complex pathogen with high antigen genomic heterogenicity due to recombination events, possessing different virulence factor expression profiles between strains, as well as complicated diverse global epidemiology of circulating Strep A serotypes. Strep A serotypes are based on variations in the major virulence factor, the M protein, with numerous variant serotypes globally and no consistent serotype observed in geographic regions or correlation with disease manifestations. At the genomic level there are even greater variations in the M-type, or *emm* type, with >200 variants. A recent genomic study showed that no single protein investigated during vaccine development has been 100% conserved between all the analysed Strep A isolates^8^. This has led to difficulty targeting a particular protein to cover all serotypes causing infections globally as a universal vaccine candidate. Gene exchange and single nucleotide polymorphisms (SNPs) causing protein sequence variation within vaccine candidates, such as within the most progressed NTD (N-terminal domain) M protein-based vaccines leads to the requirement of multicomponent inclusion to obtain acceptable levels of cross protection. Additionally broad spectrum of disease makes finding an effective long-term vaccine strategy challenging. Disproportionate Strep A disease burden and diverse serotype prevalence within LMICs, related to vaccine efficacy and protective coverage predication complicates testing when deciding on vaccine clinical endpoints generally and within these settings.

**Table 1:** Summary of studies progressing vaccines containing Group A Carbohydrate (GAC) as well as derivative GAC^PR^ (polyrhamnose) and synthetic polymers as stated.

Saccharide	Carrier protein/nanotechnology	PS activation conjugation resulting structure	Animal model	Immune response	Protection	Autoimmune	Refs.
WT GAC	TT	Periodate oxidation Chemical coupling Selective sunlike	Mouse	IgG response	60–85% Passive (Rb sera) protection in mouse IP challenge 70–80% survival in mouse IP challenge Reduced nasal colonisation	Indirect immunofluorescence – negative brain, heart, kidney	^31^
Synthetic WT GAC (6mer and 12mer)	CRM_197_	Disuccinimidyl adipate activation (6/12mer)/periodate oxidation (GAC) Chemical coupling Selective sunlike	Mouse	IgG response (12mer and WT GAC high) Antibody binding to GAS	40% OPA killing (Rb sera) 30–60% survival in mouse IP challenge	NT	^14^
WT GAC	CRM_197_						
Synthetic GAC (6mer)	TT	Cysteamine activation Chemical coupling Selective sunlike	Mouse	IgG and IgM response	NT	NT	^63^
Synthetic GAC (3mer, 6mer, 9mer)	C5a peptidase	Hydrogenation and acylation/DSG activated esters Chemical coupling Selective sunlike	Mouse	IgG response Antibody binding to GAS	30–60% OPA killing (6/9mer) 50–80& survival in mouse IP challenge	NT	^65,66^
GAC^PR^	Recombinant pneumococcal protein SP_0435	Chemical biotinylation Affinity interaction Selective	Rabbit Passive mouse	Antibody binding to GAS	Whole blood and OPA killing Survival from passive immunisation (Rb) in mouse IP challenge	Reduced ARF GlcNAc Mab reactivity Negative ELISA to human cardiac tissue lysate	^10^
GAC^PR^	Arginine Deiminase Protein	CDAP Chemical coupling Random	Mouse	IgG response Antibody binding to GAS	60% killing indirect bactericidal assay Clearance of GAS in mouse skin challenge No protection in mouse SC challenge	NT	^56^
GAC	CRM_197_	Periodate oxidation Chemical coupling Selective sunlike / random mesh	Mouse	IgG response (GAC) Significant reduction in anti-protein IgG Antibody binding to GAS No neutralisation of specific protein activity	NT	NT	^57^
	SpyCEP						
	SpyAD						
	SLO						
GAC^PR^	SpyAD	CDAP and dibenzocyclooctyne derivatization Site-direct CLICK chemistry Selective sunlike	Rabbit (sera) Mouse (challenge)	Protein IgG titres Antibody binding to GAS SLO neutralisation	OPA killing ~50% survival from passive immunisation in mouse IP challenge 100% survival from multicomponent immunisation in mouse IP challenge	Negative for antibody cross-reactivity with human heart lysates by western blot	^35^
	Non-carrier SLO and C5a peptidase						
GAC^PR^	SLO	CDAP and dibenxocyclooctyne derivatization Site-direct CLICK chemistry Selective sunlike	Mouse	IgG response	90% survival from SLO-GAC^PR^ immunisation in mouse IP challenge	NT	^62^
Synthetic GAC^PR^	Gold nanoparticles	Thiolation activation Chemical coupling Selective sunlike (star/sphere)	N/A	Competitive ELISA with anti-Rha polyclonal IgG	NT	NT	^102^
Synthetic tri-rhamnose	Ac-PADRE–lipid core	–	Mouse	IgG response	50–100% OPA killing	NT	^36^
WT GAC	GMMA	Adipic acid dihydrazide activation Chemical conjugation *Salmonella* Typhimurium GMMA	Mouse	IgG response	NT	NT	^103^
Recombinant GAC^PR^	OMV	Biological anchoring to KDO Recombinant expression *E. coli* OMV	Mouse	IgG response Antibody binding to GAS	NT	NT	^96^

Strep A can be distinguished from other beta-haemolytic streptococci species by Lancefield serotyping. This technique is based on the identification of type-specific surface exposed carbohydrates that bind to specific antibodies^9^, with *S. pyogenes* being named Strep A due to the presence of the conserved group A carbohydrate (GAC) on its surface.

### The group A carbohydrate (GAC)

Group A carbohydrate (GAC) is conserved across all Strep A strain cell surfaces^8,10^ and shown to be a key survival determinant^10^. GAC is abundant making up 40–60% of the total cell wall mass^11^, functioning to provide structural support as an environmental barrier, maintain cell morphology and enable cellular division^12^. In addition, GAC is also a major virulence determinant, providing resistance to zinc toxicity and resistance to neutrophil meditated killing^10,13,14^.

**Fig. 1:**
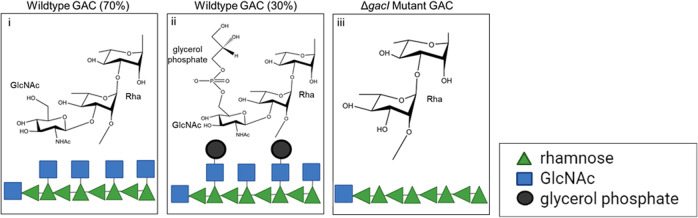
Structures of the GAC trisaccharide repeating unit. GAC is composed of a polyrhamnose backbone and GlcNAc side chains, decorated with glycerol phosphate. The wildtype repeating unit is [3)α-l-Rha*p*(1→ 2)[β-d-Glc*p*NAc(1 → 3)]α-l-Rha*p*] for 70% of the repeating units (i), and 30% modified on GlcNAc side chains with glycerol phosphate (ii) [3)-α-l-Rha*p*-(1 → 2)[β-d-Glc*p*NAc6P(S)Gro-(1 → 3)]-α-l-Rha*p*-(1 → 3)]. Δ*gacI* mutants and certain GAS strains passaged in mice have the repeating structure [3)-α-L-Rha*p*-(1 → 2)α-L-Rha*p*-(1 → 3)-α] deficient in GlcNAc sidechains (iii).

GAC polymers have an average molecular mass of 8.9 ± 1.0 kDa, corresponding to 18 repeating units^14^, though different purification methods result in varying average sizes^15^. GAC is made up of a linear polyrhamnose backbone with alternating GlcNAc sidechains, with a trisaccharide repeating unit of [3)α-l-Rha*p*(1→2)[β-d-Glc*p*NAc(1→3)]α-l-Rha*p*(1→3)]_n_ (Fig. 1)^14,16^. GlcNAc is attached to the 2-hydroxyl linked rhamnose residue^17^ extending out to the periphery from the rhamnose helix^18^. Approximately 25–30% of the GlcNAc residues on GAC polymers contain glycerol phosphate (GroP) modifications, specifically on the C6-hydroxyl group. This polysaccharide modification has remained elusive for some time and the specific function has not been fully characterised. It may, however, contribute to the immune evasion activity through resistance to zinc toxicity^19^. A Strep A mutant devoid of GlcNAc, and therefore the GroP modification, was more susceptible to killing by human whole blood, as well as in the presence of purified human neutrophils, shown mechanistically to be due to greater binding of cationic human defence peptides, cathelicidin, LL-37 to the mutant polysaccharide. This loss of GlcNAc also seems to reduce the binding of cationic bactericidal enzyme human Group IIA secreted phospholipase A2 to mutant Strep A cell surfaces^19,20^.

Branched polysaccharides composed of rhamnose and GlcNAc sidechains have been shown to be immunogenic, evidenced by rabbit and human antiserum^21,22^, computer simulations^18,23,24^ and by NMR^25^ techniques to demonstrate mAb and GAC interaction. The GlcNAc sidechain is predicted to play a role in human specific pathogenicity or immune evasion strategies. However, a consideration with using GAC as a vaccine component is the potential generation of autoimmune antibodies due to the presence of GlcNAc in many human glycan structures and similarities between GlcNAc and components of the extracellular matrix (ECM) in humans^26,27^. Clinically this has been observed with acute rheumatic fever (ARF) patients having 2- to 3- fold higher anti-GAC antibody titres at the point of infection compared to patients with pharyngitis^28^, as well as longer lived anti-GAC antibody populations in patients which have rheumatic heart disease (RHD)^29^. Isolated monoclonal antibodies from ARF patients appear to only recognise wildtype GAC, not cross reacting with mutated GAC which does not contain GlcNAc sidechains^10^, demonstrating the antibody specificity to GlcNAc epitopes. The role of GlcNAc is, however, controversial with the majority of autoimmune responses attributed instead to the M-protein in the manifestation of ARF and RHD^30^.

Antibody binding experiments have shown that GAC polymers are mainly localised to the outer surface of the cell wall^11^. This surface localisation, conservation between strains and protective properties makes GAC and derivatives of interest as a polysaccharide component of a glycoconjugate vaccine.

### GAC immunogenicity and considerations for carrier proteins

Immunological studies have revealed that GAC is accessible to antibody binding^14,31,32^, with affinity-purified anti-GAC antibodies showing opsonic properties specifically against M3, M6, M14 and M28 serotypes^32^. Host infection with Strep A induces circulating GAC specific antibodies, which may be slow to generate initially, but are thought to gradually increase with age, peaking in adolescents^33^. Some studies suggest the GlcNAc sidechain portion of the polymer^32^, particularly the branch points within the trisaccharide repeat structure, to be important in recognition and generation of opsonophagocytic IgG antibodies^22,32,34^. However, more recent data has shown antibodies also recognise rhamnose epitopes within the polymer backbone^10,35^. The entire trisaccharide of Rha-Rha-GlcNAc has been demonstrated to bind to monoclonal antibodies by several techniques including 1D-TOCSY NMR^25^. Further, that the branch structure itself is important, but GlcNAc can be substituted with rhamnose for the same response^36^. Titres of anti-GAC antibodies are thought to correlate with reduced Strep A infection incidence in adolescents compared to young children, suggesting that anti-GAC antibodies may be important in long lasting immunity to Strep A infection. This justifies the argument that carbohydrate-based antibodies are a suitable approach towards protection from disease. This concept was highlighted in a study of Mexican children which showed high titres of GAC specific IgG antibodies correlated with protection against throat carriage^31^.

Polysaccharide antigens alone are T cell independent antigens, containing a repetitive structure which can be recognised and cross-linked by B cell receptors (BCR) on B cells, but provide no T cell epitopes. This leads to B cell differentiation into plasma cells which secrete antibodies directed against the polysaccharide epitopes but with reduced immunological memory particularly in infants^37–40^. Conjugation of polysaccharide to a carrier protein enables the protein moiety to provide such T cell epitopes through presentation on MHC Class II complexes to generate CD4^+^ T cell help^41^. This strengthens and improves immune response longevity by increasing polysaccharide specific antibody levels, affinity maturation and proliferation of polysaccharide-specific B cells from memory pools, resulting in IgM to IgG isotype switching leading to higher antibody avidity^42^.

Protein carriers selected for inclusion in glycoconjugate vaccines are ideally themselves immunogenic. Suitable protein carriers must therefore enable induction of effective anti-polysaccharide immune responses and be compatible with conjugation techniques, being safe and produced at high yields and low costs^43–45^. Traditionally, carrier proteins included in currently licenced glycoconjugate vaccines were heterologous to the organism against which you wish to vaccinate^44,46^. There are currently five carrier proteins in licenced glycoconjugate vaccines; Tetanus Toxoid (TT), Diphtheria Toxoid, CRM_197_ (a non-toxic mutant of diphtheria toxin), recombinant *E. coli* produced *Haemophilus influenzae* protein D and outer membrane protein complex of serogroup B meningococcus^44,46^. These traditional carriers have been successfully included in licenced vaccines due to their compatibility with well-characterised conjugation chemistries, and their ability to induce effective long lasting anti-polysaccharide immune responses^45^. Recently, however there has been a drive for new carriers to be investigated^44^, as data suggests that repeated immunisation with the same classical carrier for different glycoconjugate vaccines in some cases dampens immunological potency and efficacy^47–50^. This is mediated by inhibition of polysaccharide antibody responses by carrier-specific B cells and suppressor T cells due to pre-existing carrier protein immunity referred to as carrier-induced epitopic suppression^49^.

Currently, the potential benefit from protective antibodies directed against the protein component has not been fully exploited. In addition to avoiding carrier-specific epitopic suppression, vaccines containing both pathogen specific polysaccharide and protein antigens provides a “double-hit” approach^45,51,52^, which may achieve broader immunity^44^. There have been several studies on conserved Strep A protein antigens as vaccine candidates, often included in multicomponent vaccines and these have potential to be utilised as glycoconjugate carrier proteins^35,53–58^. Rational protein antigen design to improve pathogen specific immune responses, in addition to providing effective T helper cell function to improve polysaccharide responses, is a key consideration for a Strep A glycoconjugate vaccine.

### Chemical conjugation for glycoconjugates

**Fig. 2:**
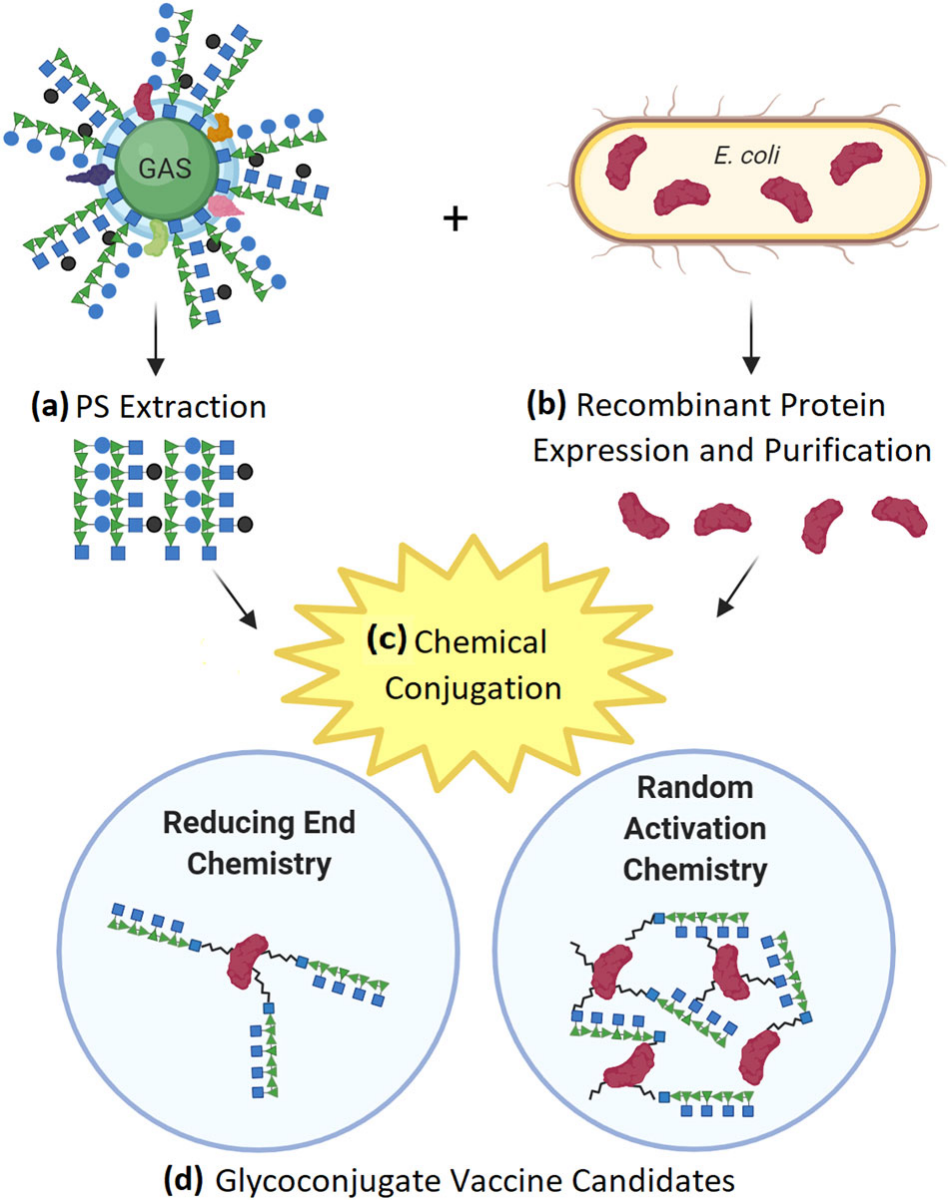
Simplified schematic representation of chemical conjugation. Polysaccharide (PS) extracted from the native organism, e.g., GAC from Strep A cells (**a**), and recombinant protein carriers expressed and purified from *E. coli* cells (**b**). Extracted PS and recombinant protein carriers containing compatible reactive groups are conjugated together using a compatible cross-linking chemical reagent (**c**) yielding heterogenous glycoconjugate vaccine candidates depending on the selected approach (**d**). Schematic shows different approaches yielding different glycoconjugate species, with reducing end chemistries leading to terminal single ended glycoconjugate products (sun-like structures), and random activation chemistries yielding cross-linked mesh-like structures of higher molecular weights with several attached protein - polysaccharide molecules.

Glycoconjugate vaccines can be synthesised through several conjugation methodologies, and the method choice is also an important consideration. This has traditionally been through chemical conjugation, which requires extraction and purification of polysaccharide directly from the organism’s cell wall or capsule against which you wish to protect. It also requires purification of recombinantly expressed protein carriers before covalent attachment of the two components can occur (Fig. 2a–c). Conjugation chemistries can utilise naturally present reactive groups or alternatively add in either cross-linking reagents to artificially introduce compatible reactive groups or chemically modify components through activation for attachment. Both these modifying reactions can improve conjugation efficiency but can also damage confirmational epitopes in the process. Chemical modification can be through reducing end selective activation, or random multiple activation along polysaccharide chains^59^ (Fig. 2d). The choice of chemical conjugation approach is often governed by structure, size, and composition of vaccine components^60^. For example, larger polysaccharides are usually randomly activated, and smaller polysaccharides activated at the reducing end to preserve protective epitopes^59^. Some selectivity can be achieved by modulating component stoichiometry^61^, targeting or introducing specific non-natural amino acids on the protein facilitating site directed attachment^62^. Such selective or milder approaches can be important for carrier proteins with a dual purpose to maintain protective B cell epitopes.

### Progress towards a GAC glycoconjugate vaccine

There have been several studies investigating GAC or components of GAC as vaccine candidates. In this section we briefly summarise key studies and advancements from selected studies, while a list of all published studies on this topic is provided (Table 1). Early studies focussed on GAC immunogenicity when conjugated to classical carrier proteins to determine the anti-polysaccharide response and the immunological epitopes for protection. These included synthetic polymers, native GAC and GAC mutated to contain no GlcNAc sidechains (GAC^PR^). The move towards modified GAC aimed to mitigate the potential risks of generating an autoimmune response^26,27^. There has been a renewed interest in GAC as a vaccine component, with studies in the last five years building on incorporation of GAC into multicomponent formulations or “double-hit” glycoconjugates. To date, these studies have all made use of chemical methods for conjugation (Table 1).

An early study by Sabharwal et al., utilised native GAC conjugated to TT and showed protection of mice from intranasal colonisation and intraperitoneal lethal challenge with two different M Strep A serotypes^31^. This study was built on by Kabanova et al. where synthetic polymers of varying lengths were conjugated to CRM_197_ to investigate size dependent immunogenicity^14^_._ These were also able to generate protective antibodies and demonstrated that a hexasaccharide (composed of four rhamnose sugars, two of which were decorated with a GlcNAc sidechain) was the minimal epitope able to invoke a robust immune response. This reiterated earlier observations that a hexasaccharide was key to the natural immune response to GAC^22^ and was further validated by a study that demonstrated equivalent immunogenicity from a hexasaccharide hapten as native GAC when conjugated to TT^63^.

To address potential vaccine safety concerns, van Sorge et al., used modified GAC devoid of GlcNAc (GAC^PR^) extracted from a genetically modified Strep A strain and conjugated to *S. pneumoniae* protein SP0435^10^. They showed that antibodies generated from the vaccine promoted phagocytic killing of multiple Strep A serotypes and protected mice against systemic infection following passive immunisation with rabbit anti-GAC antisera^10^. The conjugation methodology was unique using a Multiple Antigen Presenting System (MAPS)^64^, incorporating chemical methods for biotinylation of GAC to allow for a non-covalent bond between GAC and SP0435 containing a biotin-binding domain. This genetically mutated GAC was also able to show disease reduction with partial protection from bacteraemia and skin infections in further studies^56^, without any cross-reactivity with human heart or brain tissue lysates^35^. Further justification for modified GAC has been provided by a recent study using self-adjuvanting lipopeptides^36^. Using synthetic repeating unit epitopes this study demonstrated that the GlcNAc residue was not essential for a robust immune response to GAC. Instead, replacement with a third rhamnose could substitute for this structurally related immunogenicity, with a higher anti-GAC IgG response and equivalent or higher killing activity on Strep A strains than the wildtype repeating unit. These studies laid the foundation for interest in GAC as a viable broadly protective vaccine antigen.

The first study with a “double-hit” approach to a GAC glycoconjugate demonstrated that conjugation of modified GAC to Strep A protein ADI (arginine deiminase protein) gave a robust immune response to both components, without a loss of immunogenicity to the protein component compared with a protein alone response^56^. Following this, conjugation of synthetic polysaccharides (tri-, hexa- and nonasaccharides) to C5a peptidase (ScpA) demonstrated superior functional immunity of antibody binding to Strep A and opsonophagocytic killing over conjugation to CRM_197_ or TT with hexasaccharide haptens, and equivalent immunogenicity between carriers with a nonasaccharide^65,66^.

Recent studies have highlighted the need to preserve protein epitopes when using species specific protein carriers. This was demonstrated by di Benedetto et al., where the benefits and disadvantages of different conjugation chemistries was investigated with native GAC conjugated to CRM_197_. This revealed that random conjugation demonstrated an equivalent anti-GAC IgG response to selective conjugation but affected the anti-protein response^57^. Despite high anti-GAC IgG titres from random conjugation of GAC to three Strep A proteins SLO, SpyAD and SpyCEP, the anti-protein responses were significantly impacted and resulted in a loss of functional immunity, such as neutralisation of SpyCEP IL-8 cleavage activity. Random conjugation produces a mesh-like structure, which has been demonstrated to be effective for anti-polysaccharide responses, but likely will mask immunologically relevant protein epitopes. Selective conjugation, meanwhile, is likely to be the most effective method for “double-hit” glycoconjugates, as demonstrated with a site-specific click-chemistry approach to produce a SpyAD-GAC^PR^ conjugate^35^. In this study, four lysine residues were replaced with a non-native amino acid, p-azidomethyl phenylalanine (pAMF) in a cell-free expression system, as target sites for dibenzocyclooctyne (DMCO) derivatised modified GAC (conjugated through 1-Cyano-4-dimethyl aminopyridinium tetrafluoroborate, CDAP, chemical conjugation). IgG titres against SpyAD were high for both conjugate and protein alone, and improved shifts in antibody binding by flow cytometry for SpyAD-GAC^PR^ compared with SpyAD alone. Combination of SpyAD-GAC^PR^ with SLO and C5a peptidase resulted in improved protection in both passive and active immunisation murine challenge models compared with components alone, with no cross-reactivity observed to human heart lysates. This site-directed click chemistry approach was further investigated with SLO as a carrier protein for GAC^PR^^62^. Conjugates showed high antibody titres for GAC in all conjugates produced, and similar antibody titres for SLO in all conjugates produced compared with SLO variants alone. A specific SLO neutralising assay was not conducted to demonstrate retained functional neutralising antibodies, but an in vivo model with intraperitoneal challenge showed significant protection with the SLO-GAC^PR^ conjugate compared with immunisation with SLO and CRM_197_-GAC^PR^^62^. This was a successful demonstration that site-specific conjugation of GAC to a Strep A carrier protein provides effective antibody responses to both components and superior protection in vivo.

Though promising, the conjugates here relied on costly chemical conjugation, which has limitations such as technical challenges, low product yields, and batch to batch variation^67^. Alternative conjugation methods and vaccine structures are therefore of benefit to Strep A vaccinology to reduce costs and improve manufacturing consistency.

### Protein Glycan Coupling Technology

In recent years the development of Protein Glycan Coupling Technology (PGCT) or bioconjugation has provided an alternative and in some cases superior method to chemical conjugation^68,69^. PGCT has the potential to simplify glycoconjugate production, and several vaccines using this technology are in clinical trials^70–73^.

PGCT relies on the innate capability of certain prokaryotic cells to synthesise polysaccharides and attach them to proteins as a post-translational modification. Using PGCT the polysaccharide attachment on the carrier protein can be an asparagine (*N*-linked) or serine/threonine (*O*-linked) amino acid glycosylation^74,75^. *N*-linked glycosylation, specifically using oligosaccharyltransferase (OST) PglB from *Campylobacter jejuni* (CjPglB), is the most applied bioconjugation approach^75^. The key experiment for glycoengineering and vaccine development was the demonstration that the *pglB* operon could be cloned and fully expressed in *E. coli* cells for functional recombinant glycosylation^76^.

**Fig. 3:**
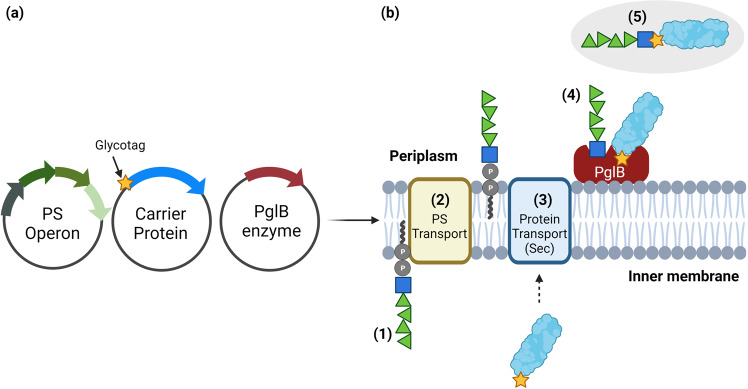
Glycoconjugate production using *C. jejuni* PglB bioconjugation inside *E. coli* host cells. **a** Plasmids containing genes encoding polysaccharide (PS) biosynthesis (green), the protein acceptor (blue) containing a glycotag (yellow star), and the oligosaccharyltransferase (OST) PglB (red) are co-transformed into *E. coli* host cells. **b** Bioconjugates are produced as follows; (1) The PS biosynthesis locus is expressed and built onto undecaprenol-pyrophosphate (Und-PP) lipid linkers within the inner membrane. (2) The PS is flipped from the cytoplasm to the periplasm by a specific flippase enzyme. (3) Synthesised carrier proteins are exported to the periplasm through the Sec secretion system. (4) In the periplasm both the PS, and the carrier protein containing a specific glycotag can be recognised by the PglB OST enzyme. PglB transfers the PS from Und-PP onto the asparagine residue within the glycotag D/E-X-N-X-S/T motif on the fully folded carrier protein, resulting in protein glycosylation. (5) An inexhaustible supply of glycoproteins can be subsequently purified from *E. coli* cells.

Polysaccharides for exploitation of this system, are first synthesised onto an undecaprenol-pyrophosphate (Und-PP) lipid linker attached to the cytoplasmic leaflet of the inner membrane. A flippase enzyme is then responsible for the translocation of the fully synthesised polysaccharide across to the periplasm, where it can subsequently be recognised by PglB or other OST and attached onto a recognition sequon on a given acceptor protein (Fig. 3)^77,78^. The protein recognition sequon for *N*-linked glycosylation is D/E-X-N-X-S/T, where X represents any amino acid except proline, and positive D/E and S/T amino acids are at the ±2 positions, pivotal in locating asparagine (N) as the acceptor amino acid^79^. Unlike eukaryotic glycosylation, bacterial *N*-linked glycosylation occurs after protein folding, therefore to be accessible to PglB engineered Glycotags of the D/E-X-N-X-S/T sequon can be added to the N- and C- termini of carrier proteins allowing enhanced glyco-modification in vaccine design^80,81^.

PGCT is a feasible alternative to chemical conjugation^78^, with benefits such as *E. coli* systems producing inexhaustible fully synthesised recombinant polysaccharide resources, and readily purified glycoproteins at reduced costs and improved yields^82^. This great promise is currently limited by PglB specificity, such that only polysaccharides with a reducing end containing an acetamido group at the C2 position are permissive for wildtype enzyme transfer^79,83^. However, this limitation can be mitigated by modification of the PglB enzyme by directed evolutionary mutagenesis to improve transfer compatibility and efficiency^84–86^.

Alternative OST enzymes are also widely used for *N*-linked glycosylation^74,77,87,88^, and *O*-linked OST enzymes, such as PglL and PglS for glycans with galactose^89,90^ and glucose end groups^91,92^. As the discovery and understanding of bacterial OSTs increases there will be further opportunities to develop PGCT for custom designed glycoconjugate vaccines such that theoretically almost any glycan could be coupled to any protein. In addition, PGCT provides the opportunity to couple multiple glycans at precise positions on a given carrier protein, which has been demonstrated to increase glycoconjugate vaccine efficacy^93^.

Furthermore, PGCT offers benefits such as minimal alteration to the protein carrier and target recombinant polysaccharide, dissimilar to chemical conjugation approaches which are often harsher in their attachment method.

### GAC biosynthesis and exploitation of recombinant polyrhamnose structures

**Fig. 4:**
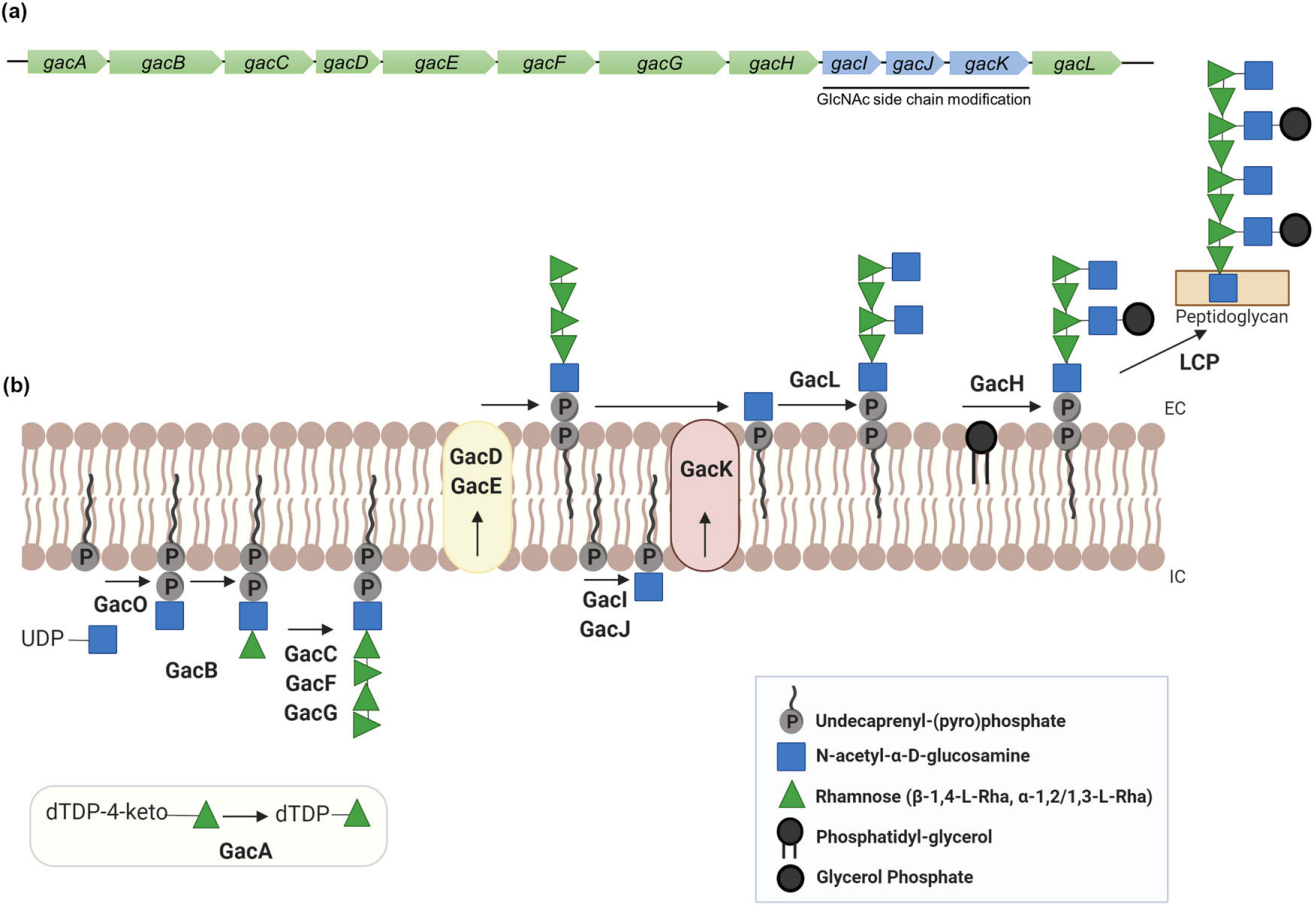
The gac operon in Strep A produces GAC containing a polyrhamnose backbone decorated with alternate GlcNAc side chains. **a** Schematic representation of the *gac* operon (*gacA-L*) in Strep A. Horizontal arrows represent each gene designation with colour denoting predicted gene function. Green, polyrhamnose biosynthesis; blue, GlcNAc biosynthesis. **b** Schematic diagram of GAC biosynthesis. GAC biosynthesis is initiated on lipid linked GlcNAc attached to the inner leaflet of the periplasmic membrane for polyrhamnose synthesis catalysed by rhamnosyltransferase enzymes (GacBCFG). After polymerisation, the polyrhamnose backbone is flipped to the outer leaflet by an ABC transporter (GacDE complex) before GlcNAc (GacL) and glycerol phosphates (GacH) are transferred to the polyrhamnose backbone as a sidechain modification. A LytR-CpsS-Psr (LCP) phosphotransferase protein is hypothesised to attach GAC to peptidoglycan via a phophodiester bond^17^.

GAC polymers are encoded by a conserved 12-gene cluster termed *gacA-L*^8,10^ (Fig. 4a). The operon is highly conserved with one study finding that 2017 of 2083 tested Strep A genomes had >70% DNA sequence similarity for the entire 12-gene cluster^8^ supporting observations from a smaller dataset^10^. Within the operon the first seven genes (*gacA-G*) encode biosynthesis of the polyrhamnose backbone, conserved across other streptococci groups, specifically A, B, C and G^94^, whilst 3 genes (*gacI-K*) are known to be implicated in GlcNAc sidechain attachment and modification^10^. To date not all genes have been fully characterised, and some are believed not to be essential to survival due to frameshift mutations in some Strep A genomes^8,13^. However, the rhamnose encoding genes^10^ and the availability of l-rhamnose substrates^12^ have been shown to be essential to Strep A survival.

There is a strong case for the exploitation of GAC with PGCT. A rhamnose backbone structure related to GAC in *Streptococcus mutans* has previously been expressed and exported to the surface of *E. coli* cells, altering the LPS profiles, with similarity shown between native rhamnose biosynthesis pathways in *S. mutans* and the reconstituted pathways expressed and synthesised in *E. coli* host cells^95^. Recently, Castro et al followed a similar recombinant approach, where *E. coli* cells encoded the genes for GAC rhamnose backbone biosynthesis and produced Outer Membrane Vesicles (OMVs) loaded with the carbohydrate^96^. Their work has shown that the GAC rhamnose backbone can be built successfully in *E. coli* with the correct chemical structure and produces antibodies in mice and rabbits that can target Strep A serotypes. This system can now serve as a foundation for PGCT with Strep A antigens.

GAC has a GlcNAc at the reducing end, suggesting it would be suitably recognised by *Cj*PglB, however the following sugar in the chain, rhamnose, is attached to the GlcNAc by a β-1,4 linkage by GacB^94^. This particular linkage may not be recognised or transferred by *Cj*PglB efficiently, as demonstrated by studies assessing *Cj*PglB transfer capability of a GlcNAc β-1,4 GlcNAc disaccharide attached to either a non-native eukaryotic isoprene lipid linker^97^, or a native Und-PP lipid linker^98,99^. Therefore, alternative strategies may be required to transfer Strep A rhamnose polymers by PGCT.

### Concluding remarks

The global imperative to develop a Strep A vaccine has resulted in a burgeoning field of GAS targeted glycoconjugate vaccines. Following initial studies demonstrating a robust immune response to GAC, focus has shifted to “double-hit” glycoconjugates incorporating species specific carrier proteins along with GAC, either native or modified to remove GlcNAc side chains. Studies have suggested that milder selective approaches are more appropriate for “double-hit” glycoconjugates to prevent shielding of protein immune epitopes, as they are less likely to disrupt the protein structure and conformation, ensuring its role as a T cell epitope carrier, as well as a protective antigen itself. However, such approaches may reduce overall polysaccharide attachment due to limiting the region of the chain which is activated and available for attachment^100^. This in some respects is similar to bioconjugation where protein carriers are engineered with glycotags at the N- and C- termini, which is more likely to preserve the protein’s stability and structure, and therefore B and T cell epitopes^81^. Investigation of the use of bioconjugation for generation of a Strep A glycoconjugate would help lead the way to a cost-effective Strep A vaccine.

Studies with Generalised Modules for Membrane Antigens (GMMA) showed that different mechanisms of presentation, specifically the structure and chain length of the polysaccharide antigen, dictates the dominance of T cell responses. Based on a *S. typhi* capsule glycoconjugate, shorter polysaccharide antigens appear to require more T cell help for robust immune stimulation compared to glycoconjugates containing longer polysaccharide antigens^101^. Therefore, carrier proteins which can strongly stimulate T cell help responses may be necessary for future vaccines with GAC. Such T cell activity is important in the magnitude as well as response duration, as without T cell activity apoptosis of polysaccharide-specific B cells in the spleen and depletion of polysaccharide-specific B cells in the bone marrow can occur leading to hypo-responsiveness^101^. Such impact of polysaccharide size on B and T cell interactions is known to be antigen-specific^41,100^, requiring future studies to focus on deciphering the best protein carrier to induce T cell activity and enable GAC directed memory responses.

There has also been increasing interest in novel non-protein carriers for GAC. Gold nanoparticles have been shown to successfully present GAC for recognition by immune sera, though its own intrinsic immunogenicity was not assessed^102^. Self-adjuvating lipopeptides have shown great promise with GAS peptides and have also shown a robust immune response against GAC^36^, with recent studies taking this further showing strong immune responses to conjugated GMMA^103^ and recombinant OMVs^96^. Such technologies will help to build up an understanding of a protective immune response to GAC and derivatives, and push for a low-cost solution to the global need for a vaccine. Recombinant technology, particularly PGCT, may answer this need with its low-cost, inexhaustible and renewable supply along with its ability for rational design^82^. Carrier proteins and glycans can easily be manipulated such that glycosylation sites can be precise, glycan number increased as needed and chain lengths potentially controlled.

In summary, GAC is a conserved antigen, found in all isolated Strep A strains, a property which recommends it for a universal global vaccine against Strep A infection. Investigation into cost-effective solutions for glycoconjugate production is imperative for a global vaccine, particularly to target low- and middle-income countries. Newer technologies such as PGCT and GMMA/OMVs show great promise towards this aim and examination with GAC is warranted. Impressive progress has been made towards a Strep A glycoconjugate vaccine, and further investigation into production of “double-hit” conjugates and their protection from infection would be a sweet reward.
